# Large Platelet and Endothelial Extracellular Vesicles in Cord Blood of Preterm Newborns: Correlation with the Presence of Hemolysis

**DOI:** 10.3390/diagnostics11081316

**Published:** 2021-07-22

**Authors:** Andrea Hujacova, Jan Sirc, Kristyna Pekarkova, Tereza Brozova, Marie Kostelanska, Jakub Soukup, Tibor Mosko, Karel Holada, Zbynek Stranak

**Affiliations:** 1 First Faculty of Medicine, Institute of Immunology and Microbiology, Charles University, General University Hospital in Prague, Studnickova 7, 12820 Prague 2, Czech Republic; ajka.hujacova@gmail.com (A.H.); vrbova.k@gmail.com (K.P.); marie.kostelanska@lf1.cuni.cz (M.K.); jakub.soukup@lf1.cuni.cz (J.S.); tibor.mosko@lf1.cuni.cz (T.M.); 2 Department of Neonatology, Institute for the Care of Mother and Child, Podolske Nabrezí 157/36, 14700 Prague 4, Czech Republic; jan.sirc@upmd.eu (J.S.); tereza.brozova@upmd.eu (T.B.); zbynek.stranak@upmd.cz (Z.S.)

**Keywords:** flow cytometry, immune electron microscopy, cord blood, extracellular vesicles

## Abstract

Different biomarkers are investigated to detect the causes of severe complications in preterm infants. Extracellular vesicles (EVs) are recognized as an important part of cell-to-cell communication, and their increased levels were reported in numerous pathological states. We aimed to increase our knowledge about the incidence of platelet and endothelial EVs in cord blood of preterm newborns using conventional flow cytometry. The presence of platelet (CD36+CD41+), activated platelet (CD41+CD62+), and endothelial (CD31+CD105+) EVs was analyzed. Immune electron microscopy was used to confirm the presence of EVs and the specificity of their labeling. The size of detected extracellular vesicles was in the range 400–2000 nm. The differences in the counts of EVs between the preterm and control group were not significant and no correlation of EVs count with gestation age was recorded. Cord blood plasma samples with free hemoglobin level > 1 mg/mL had more than threefold higher counts of CD36+CD41+ and CD41+CD62+ EVs (*p* < 0.001), while the count of CD31+CD105+ EVs was only moderately increased (*p* < 0.05). Further studies utilizing cytometers with improved sensitivity are needed to confirm that the analysis of large platelet and endothelial EVs mirrors the quantitative situation of their whole plasma assemblage.

## 1. Introduction

Preterm birth represents a serious challenge for perinatal medicine contributing to over 70% of perinatal mortality in developed countries. Surviving infants frequently suffer from cardiorespiratory problems and neurodevelopmental impairments [1]. The most common cause of preterm birth is infection associated with intra-amniotic microbial invasion and chorioamnionitis [2]. The release of endotoxins and exotoxins from bacteria colonizing choriodecidual space initiates production of inflammatory cytokines, starting the maternal and fetal inflammatory response. The infiltration of colonized tissues with cytokine producing immune cells and invasion of bacteria into the amniotic cavity further augment the distress of the fetus. Fetal inflammatory response syndrome (FIRS) is defined by increased systemic inflammatory cytokine concentrations, funisitis, and fetal vasculitis. Multiorgan involvement in FIRS has been demonstrated in the hematopoietic system, thymus, adrenal glands, skin, kidneys, heart, lung, and brain [3]. Biological markers including C-reactive protein, procalcitonin and interleukin-6 levels in cord blood can be used to attempt to identify fetal inflammation, but their specificity is limited [3,4]. Histological evaluation of placenta tissue for infiltrating neutrophils seems to be a more specific but impractical approach due to the long turnaround time of the test results.

Recently, the analysis of extracellular vesicles (EVs) in body fluids, also called liquid biopsies, represents an attractive opportunity to complement the existing diagnostic tools. The main categories of EVs secreted by cells are exosomes, microvesicles, and apoptotic bodies. They are all vesicles with a bilayer lipid outer membrane, but they differ in biogenesis, size, and associated cargo [5]. EVs participate in cell communication and are involved in many physiological and pathological processes including pregnancy and systemic inflammatory response [6]. A promising strategy in employing the diagnostic potential of EVs represents the analysis of their nucleic acid cargo, which benefits from high specificity and sensitivity of molecular biology methods. Another approach is an immunophenotype quantitative analysis of EVs using flow cytometry, analogous to the analysis of leukocyte subsets in the blood. The widespread availability of clinical flow cytometers, straightforward procedure, and fast handling of large volume of samples make flow cytometry particularly attractive for clinical EVs analysis. However, studies on EVs are complicated by their heterogeneity and small size, which makes their analysis prone to various artifacts [7]. The conventional flow cytometers are optimized for the analysis of blood cells and allow detecting only a small population of the largest EVs, while the most numerous small EVs are below their sensitivity [8]. At the same time, even the analysis of this small population of large EVs may have diagnostic potential, as is suggested by various studies reporting increased numbers of EVs in different body liquids and disease states [9]. The most abundant EVs in blood plasma are of platelet origin, but endothelial and leukocyte EVs were also frequently analyzed.

Thus far, flow cytometry studies on EVs in cord blood have mostly focused on preeclampsia patients where their analysis seems to provide information about the ongoing placenta damage and platelet activation [10]. Spontaneous delivery is likely associated with increased levels of cord blood EVs in comparison with cesarean section, probably due to cellular stress during the labor [11,12]. Flow cytometry studies of cord blood EVs in preterm newborns are rare. Wasiluk et al. reported a higher percentage of platelet-derived EVs in preterm newborns [13]. In our present study, however, comparing a small number of preterm and term patients, we did not find a significant difference in the counts of platelet EVs [14]. This was an intriguing result as preterm birth is associated with both higher morbidity and gestation age dependent changes in the expression of platelet receptors [15], which both, in theory, may affect EV counts.

To learn more about the presence of large platelet and endothelial EVs in cord blood of preterm newborns and about the utility of conventional flow cytometry in their analysis, we accomplished a blinded study incorporating diverse control measures to improve its reliability.

## 2. Materials and Methods

### 2.1. Study Population

A prospective cohort study was conducted in a single tertiary neonatal intensive care unit (NICU). Inclusion criteria were: indicated Cesarean section ≤34 weeks of gestation in study group (PRTM, *n* = 20) and planned Cesarean section after 36 weeks of gestation in control group (CTRL, *n* = 10). Cases with insufficient volume of umbilical blood were excluded. The study protocol (NV17-31403A) was approved by the ethical committee of Institute for the Care of Mother and Child at 02JUN2015 (No. 2015/06-02-4). Written informed consent for laboratory analysis and anonymous use of clinical data was obtained from the parents of each infant. Table 1 presents a description of the experimental cohorts.

### 2.2. Obstetrical Care and Umbilical Cord Blood Sampling

Gestational age was established using first-trimester ultrasound in all cases. Fetal growth restriction (FGR) was diagnosed prenatally by obstetricians using 2D and Doppler measurement of the fetus (abdominal circumference, estimated fetal weight, and end-diastolic flow patterns in the umbilical and uterine artery) [16].

Antenatal steroids (four 8 mg doses of dexamethasone administered intramuscularly every 12 h) were used before anticipated preterm birth in all cases. A single course of dexamethasone complied with recommendation for pregnant women between 24 0/7 weeks and 33 6/7 weeks of gestation who are at risk of preterm delivery within 7 days, including for those with ruptured membranes and multiple gestations [17].

Preterm premature rupture of membrane (PPROM) was diagnosed by examination with a sterile speculum to verify the leakage of amniotic fluid into the vagina. In clinically unclear situation, the measurement of insulin-like growth factor binding protein in vaginal fluid was used (ACTIM PROM test; MedixBiochemica, Kauniainen, Finland) to confirm the diagnosis of PPROM [18].

Caesarean section was performed by Pfannenstiel technique. Placentas were delivered manually with cord traction and immediately transported to the adjacent blood collection room. Venous cord blood (4 mL) was collected into a syringe containing 1 mL of ACD-A solution (Anticoagulant Citrate Dextrose Solution) using 20G needle. The blood was transferred to 13 mL polypropylene tube (TPP #91016) and immediately transported to the Department of Clinical Biochemistry for further processing.

The placenta, fetal membranes, and umbilical cord were fixed in 4% buffered formaldehyde. The degree of polymorphonuclear leukocyte infiltration was assessed according to the Amsterdam classification. Inflammatory changes in chorionic vessels, umbilical vein, and/or umbilical artery (or arteries) were considered as fetal inflammatory response [19].

Fetal inflammatory response syndrome (FIRS) was defined an umbilical interleukin 6 concentration higher than 11.0 ng/L and/or the presence of histopathologically proven fetal inflammatory response (funisitis and chorionic plate vasculitis) [20].

### 2.3. Laboratory Measurements

Serum IL-6 level was assessed by electrochemiluminescence immunoassay (Cobas 6000, e601 module, Roche Diagnostics, Mannheim, Germany). The upper detection limit of IL-6 was 5000 ng/L (range 0–5000 ng/L). Immunoluminometric assay (Lumitest PCT, Brahms, Germany) was used for procalcitonin (PCT) analysis. Luminescence was measured automatically in a Berilux Analyser (Behring Diagnostics, Marburg, Germany). The upper detection limit of PCT was 100 µg/L. C-reactive protein (CRP) was measured by immunoturbidimetry (Cobas 6000, c501 module, Roche Diagnostics, Mannheim, Germany). The volume of blood required for analysis was 0.6 mL.

### 2.4. Neonatal Morbidity

Neonatal outcomes (respiratory distress syndrome, patent ductus arteriosus, intraventricular hemorrhage, necrotizing enterocolitis, bronchopulmonary dysplasia, retinopathy of prematurity, and periventricular leukomalacia) were followed up according to the Vermont Oxford definition [21]. Composite morbidity included periventricular leukomalacia, intraventricular hemorrhage, bronchopulmonary dysplasia, and death.

### 2.5. Cord Blood Processing

Anticoagulated venous cord blood was centrifuged at 2800× *g* (15 min, 24 °C) in a swing out rotor and the top 2 mL of platelet poor plasma (PPP) was transferred to fresh 13 mL tube and centrifuged again at 2800× *g* (15 min, 24 °C) to remove residual platelets. The sample of platelet free plasma (PFP) was divided into two 950 µL aliquots in cryogenic vials (T311-1, Simport Scientific Inc., Beloeil, Québec, Canada), flash frozen in liquid nitrogen, and stored at −80 °C. All collected samples were labeled by the attending physician and blinded for further laboratory analysis.

### 2.6. Antibodies

The following fluorescently labeled monoclonal antibodies (MAb) against platelet or endothelial markers and corresponding isotype controls were purchased from Exbio (Vestec, Czech Republic) and used in the final concentrations: 3.5 µg/mL CD31 FITC (IgG1, clone MEM-05), 0.25 µg/mL CD36 FITC (IgG1, clone TR9), 2 µg/mL CD41 PE (IgG1, clone MEM-06), 2.5 µg/mL CD41 FITC (IgG1, clone MEM-06), 0.5 µg CD62P PE (IgG1, clone AK4), 1.5 µg/mL CD105 PE (IgG2a, clone MEM-226) and isotype controls 3.5 µg IgG1 FITC (clone MOPC-21), 2 µg/mL IgG1 PE (clone MOPC-21), and 1.5 µg/mL IgG2a PE (clone PPV-04). All MAbs were titrated [22] to estimate the saturating concentration using anticoagulated blood of healthy donor or using cultured HUVEC cells. The polyclonal goat anti-mouse IgG antibody labeled with 10 nm colloidal gold was purchased from Sigma-Aldrich (cat.n. G7652).

### 2.7. Labeling of Extracellular Vesicles

The aliquot of PFP was thawed on ice, mixed, and 40 µL of PFP was pipetted into 1.5 mL Eppendorf tubes containing 10 µL of antibody mixtures CD36 FITC/CD41 PE, CD41 FITC/CD62 PE, and CD31 FITC/CD105 PE. The labeling with MAbs was carried out in two independent replicates. Samples were incubated in the dark on ice for 30 min. The unbound MAbs were washed away by 1 mL of ice-cold PBS with 0.1% BSA (PBS-BSA) and EVs spun down at 15,000× *g* (20 min, 4 °C). The pellets were resuspended in 300 µL of ice-cold PBS-BSA and immediately analyzed using flow cytometry. The PBS-BSA was filtered with 100 nm filter.

### 2.8. Flow Cytometry

**Figure 1:**
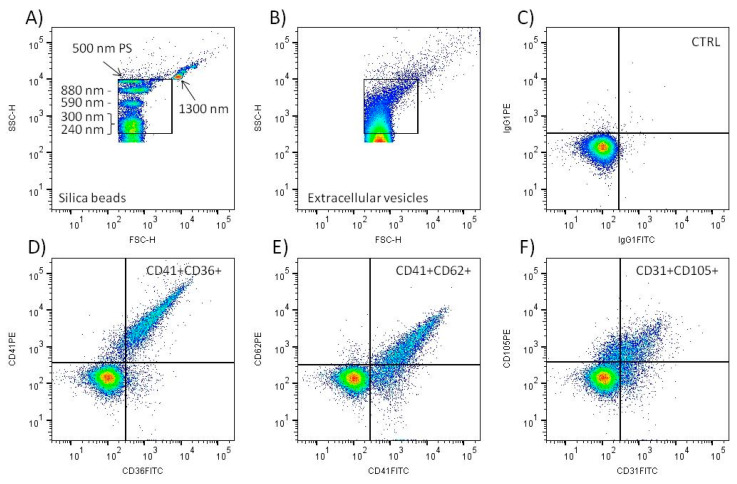
Flow cytometry analysis of large EVs in cord blood plasma. Standard silica and polystyrene (PS) beads were utilized to create EV gate in the SSC-H/FSC-H scattergram (**A**). Typical scattergram signal of cord blood plasma extracellular vesicles (**B**). Only events inside the gate were analyzed. Representative density plot of negative control sample (CTRL) labeled with isotype IgG1 control MAbs (**C**). Representative density plots of samples labeled with CD41 PE + CD36 FITC (**D**), CD41 FITC + CD62 PE (**E**), and CD31 FITC + CD105 PE (**F**) MAbs. Only double fluorescence positive events in the upper right quadrant were included in the analysis.

The samples were analyzed using BD FACSCantoTM II flow cytometer (BD Biosciences, San Jose, CA, USA) equipped with 633, 488, and 405 nm lasers. The voltages were set on FCS 555 V, SSC 500 V, FL1 500 V, and FL2 600 V (all on 488 nm laser). The trigger threshold of the cytometer was set on SSC value of 200 (488 nm laser). The performance of the cytometer was checked daily using calibrating CST beads (BD Biosciences, East Rutherford, NJ, USA). The data were recorded uncompensated by BD FACSDiva Software version 6.1.2 (BD Biosciences, San Jose, CA, USA). ApogeeMix beads (Cat#1493, Apogee Flow Systems, Northwood, UK) were used daily to define EVs gate (Figure 1) as described previously [14]. The mixture contains 180, 240, 300, 590, 880, and 1300 nm silica beads with refractive index η = 1.43 and 110 and 500 nm fluorescent latex beads with refractive index η = 1.59. The samples were analyzed at low speed (~10 µL/min) and the events collected for 2 min. Compensation for spectral overlap was done using blood platelets labeled with CD36 FITC/CD41 PE MAbs and the compensation matrix created using FlowJo software version 10.6.0 (BD Biosciences, Ashland OR, USA).

### 2.9. Concentration of Free Hemoglobin in Blood Plasma

Hemoglobin (Hb) concentration was determined spectrophotometrically. The absorption spectrum of PFP (320–630 nm) was recorded in a 1 mm glass cuvette using the Eppendorf BioSpectrometer^®^ (Eppendorf Czech & Slovakia s.r.o, Ricany u Prahy, Czech Republic) and the concentration calculated as described previously [23]. Samples with a Hb concentration above 0.1 mg/mL were marked as hemolytic.

### 2.10. Data Evaluation and STATISTICS

Flow cytometry data were analyzed using FlowJo™ software (BD Biosciences, Ashland OR, USA). The count and median fluorescence intensity of double positive particles recorded in the EV gate were evaluated (Figure 1). The data represent the mean value of two independently labeled sample aliquots. The count of double positive events after labeling with isotype control MAbs was subtracted. Calibration of SSC signal to standard units (nm) was done using ApogeeMix beads (Apogee Flow Systems, Northwood, UK) and FCMPASS Software as described previously [24]. Statistical analysis was performed using GraphPad Prism software version 5.03 (GraphPad Software, San Diego, CA, USA). Student’s T-test or Mann–Whitney U test were utilized for comparison of data where appropriate. The differences between groups with *p* < 0.05 were assumed significant.

### 2.11. Immune Electron Microscopy

The EV pellet sedimented by 15,000× *g* spin was resuspended in 50 µL of PBS and fixed by equal volume of 2% paraformaldehyde (30 min on ice). The reaction was stopped by 200 µL of 0.5 M glycine in PBS with 1% BSA (5 min on ice), EVs sedimented (15,000× *g*, 20 min, 4 °C), and resuspended in 100 µL of PBS-BSA. The EVs were labeled by MAb CD41 PE (20 µg/mL, 30 min on ice) and washed with 1 mL of PBS-BSA (15,000× *g*, 20 min, 4 °C). The pellet was resuspended in 25 µL of PBS-BSA and 10 µL applied on formvar carbon coated nickel grids (Electron Microscopy Sciences, Hatfield, PA, USA). After 20 min the grid was washed by PBS with 5% BSA and incubated with colloidal gold conjugated polyclonal antibody against mouse IgG (1:10 dilution, 30 min). After extensive washing the sample was postfixed with 1% glutaraldehyde, washed again, and contrasted with 2% uranyl acetate. The samples were observed using transmission electron microscope (TFS Morgagni 268, Thermo Fisher, Waltham, MA, USA) equipped with MegaView III CCD camera (Olympus, Shinjuku, Japan).

## 3. Results

### 3.1. Characteristics of Study Groups

Overall, 20 preterm infants (PRTM group) and 10 controls (CTRL group) were enrolled into the study. Characteristics of the study groups are expressed in Table 1. All PRTM and no CTRL patients were treated with antenatal steroids before the delivery. The most common causes leading to preterm birth were preterm premature rupture of membrane (PPROM), fetal growth restriction (FGR), and fetal inflammatory response syndrome (see Table 1). Neonatal morbidity of the PRTM group included respiratory distress syndrome (*n* = 13), patent ductus arteriosus (*n* = 2), intraventricular hemorrhage (*n* = 2), bronchopulmonary dysplasia (*n* = 2), spontaneous intestinal perforation (*n* = 1), and retinopathy of prematurity (*n* = 1). The composite morbidity was found in 3 out of 20 PRTM patients. No neonatal complications were presented in seven PRTM infants and all patients in the CTRL group.

### 3.2. Validation of Flow Cytometry EVs Detection

The EV gate was set to include the signal of 240, 300, 590, and 880 nm silica and 500 nm polystyrene beads on the scattergram (Figure 1A). The signal of 110 nm polystyrene and 180 silica beads was below the sensitivity of the flow cytometer. The signal of 1300 nm silica bead was left outside the EV gate. This definition of EV gate excludes the signal of intact blood platelets, as we demonstrated previously [14]. The beads data and FCMPASS software were utilized to calculate the approximate size of EVs detected inside the gate (Figure 1B). The calculation used “high EV refractive index” provided in the software [24]. The calculated size of the EVs was in the range 400–2000 nm. The acquisition of the samples proceeded at a speed less than 500 events per second, and only three samples had to be diluted 1:1 to lower the speed to meet the limit. The average acquisition speed of all analyzed tubes was 268 ± 127 events/s (median = 219, *n* = 360). The count of particles detected in the EV gate present in PBS-BSA buffer was ~10 times lower (622 ± 91/µL) than in the non-labeled plasma samples (6064 ± 2948/µL). Negative control samples labeled with IgG isotype control MAbs displayed a minimal number of double positive events (Figure 1C). In comparison, the labeling of plasma samples with platelet and endothelial MAbs led to the detection of a significant number of double positive EVs (Figure 1D–F).

### 3.3. Confirmation of EVs Presence in Cord BLOOD Plasma by Immune Electron Microscopy

**Figure 2:**
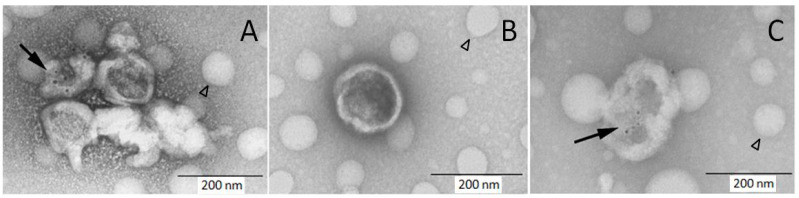
Immune electron microscopy demonstrates the presence of platelet EVs in cord blood plasma. Samples were labeled with CD41 PE MAb and secondary antibody conjugated with colloidal gold followed by staining with uranyl acetate. Some of visualized EVs are decorated with colloidal gold ((**A**,**C**) black arrow) and some are non-labeled (**A**,**B**). The round hollow structures are artifacts present on carbon grids (arrowhead).

The EV pellet was prepared similarly as for flow cytometry analysis. Labeling of samples with CD41 antibody and colloidal gold conjugated secondary antibody demonstrated the presence of platelet EVs (Figure 2). Most detected EVs appeared as “cup-shaped” particles with size in the 150–300 nm range, and only a portion of them was decorated with colloidal gold.

### 3.4. Counts of Platelet End Endothelial Large EVs in Cord Blood Plasma

**Figure 3:**
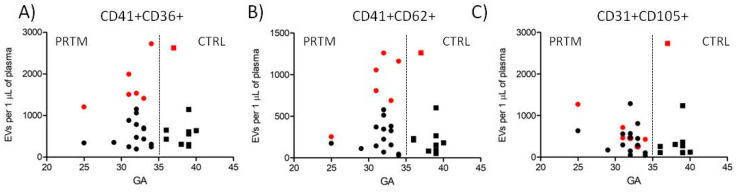
Counts of cord blood plasma large EVs plotted against newborn gestational age. (**A**) Platelet (CD41+CD36+), (**B**) activated platelet (CD41+CD62+), and (**C**) endothelial (CD31+CD105+) EVs in preterm newborns (PRTM; circles, *n* = 20) and control newborns (CTRL; squares, *n* = 10). Gestational age (GA) is in weeks. Red symbols represent hemolytic samples (Hb > 0.1 mg/mL).

Platelet and endothelial EVs were defined as events inside the EV gate and double positive for the cell specific markers (Figure 1). Agreement between the counts of EVs in the two independently labeled sample aliquots was satisfactory. Within 90 measured aliquot pairs, 69 (76.7%) had a difference from the mean count less than 10% (average 4.4 ± 2.9%), and, out of 21 pairs with a difference above 10% (average 13.6 ± 4.4%), only one pair differed more than 20% (29.2%). No significant differences in the count of platelet (CD41+CD36+), activated platelet (CD41+CD62+), and endothelial (CD31+CD105+) EVs between PRTM and CTRL groups were identified. The number of detected EVs in individual samples was relatively low, ranging from less than 100 to almost 3000 EVs/µL of plasma. Platelet EVs were approximately twice as much numerous as activated platelet and endothelial EVs (Figure 3 and Table 2). The counts of studied large EVs did not correlate with gestational age at delivery. Samples with hemoglobin concentration >0.1 mg/mL (*n* = 7) displayed notably higher counts of platelet and activated platelet EVs than the rest of the samples (Figure 3). Exclusion of the hemolytic samples from the comparison of EV counts did not lead to significant differences between PRTM and CTRL groups).

### 3.5. Comparison of EV Counts Present in Hemolytic and Non-Hemolytic Cord Blood Plasma Samples

**Figure 4:**
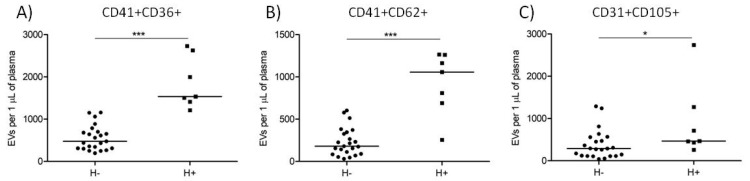
Counts of large EVs in hemolytic (H+) and non-hemolytic (H−) cord blood plasma samples. Platelet ((**A**) CD41+CD36+), activated platelet ((**B**) CD41+CD62+), and endothelial ((**C**) CD31+CD105+) EV counts in hemolytic (H+; squares, *n* = 7) and non-hemolytic (H−; circles, *n* = 23) samples. The medians are shown. * *p* < 0.05, *** *p* < 0.001.

Out of 30 analyzed plasma samples, 6 PRTM and 1 CTRL samples had concentrations of hemoglobin > 0.1 mg/mL and were labeled as hemolytic (*n* = 7). The rest of the samples were labeled as non-hemolytic (*n* = 23). The concentration of plasma hemoglobin was 0.175 ± 0.057 and 0.053 ± 0.018 mg/mL in the hemolytic and non-hemolytic groups, respectively (*p* < 0.001). Counts of platelet and activated platelet EVs were significantly higher in the hemolytic samples while counts of endothelial EVs were just moderately elevated (Figure 4 and Table 3).

### 3.6. Comparison of Cord Blood Plasma Large EVs Fluorescence Intensity

**Figure 5:**
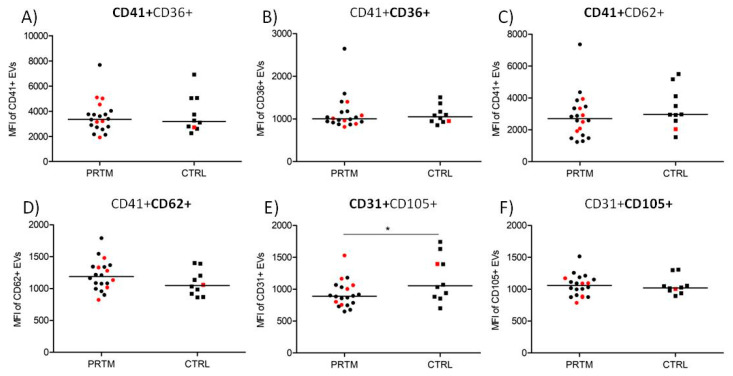
Comparison of median fluorescence intensity of labeled large EVs in cord blood plasma of PRTM and CTRL. Platelet (CD41+CD36+), activated platelet (CD41+CD62+), and endothelial (CD31+CD105+) EVs in preterm newborns (PRTM; circles, *n* = 20) and control newborns (CTRL; squares, *n* = 10) were analyzed. MFI of EVs labeled with CD41 PE (**A**), CD36 FITC (**B**), CD41 FITC (**C**), CD62 PE (**D**), CD31 FITC (**E**), and CD105 PE (**F**) are presented. Red symbols represent hemolytic samples (Hb > 0.1 mg/mL). The medians are shown. * *p* < 0.05.

The median fluorescence intensity (MFI) of CD31-labeled EVs was significantly higher in CTRL group than in PRTM patients (1166 ± 335 vs. 926 ± 203; *p* < 0.05). The differences after labeling with CD36, CD41, CD62, and CD105 MAbs were not significant (Figure 5). Only double positive large EVs were analyzed (Figure 1D–F). The MFI values of EVs in hemolytic and non-hemolytic samples were similar.

## 4. Discussion

### 4.1. Justification of the Study Layout

Flow cytometry is frequently used to demonstrate increased numbers of plasma EVs of platelet or endothelial origin in various pathologies connected with inflammation, infection, thrombosis, and cancer [9]. Although neonatology is in urgent need of new disease biomarkers, studies on EVs in newborns are extremely rare [13,14]. Thus far, no study quantitatively compared counts of platelet and endothelial EVs in cord blood of preterm and term newborns. Hyporeactivity of blood platelets and different expression of platelet receptors in preterm newborns is well documented. For example, the expression of both CD41 and CD62P was shown to be lower in preterm newborns [15]. Similarly, the impairment of vascular endothelium function and expression of several key endothelial receptors seems to be gestational age dependent [25]. How these discrepancies affect the numbers of detected EVs, and the possible diagnostic potential of flow cytometry analysis of EVs in cord blood is not known.

### 4.2. Experimental Design, Preparation of Samples and Detection of EVs

To gain basic knowledge about the feasibility of EV analysis, we designed our study to include a cohort of preterm newborns differing in clinical outcomes and a control cohort of border late preterm and term healthy newborns. A variety of preanalytical parameters may affect the results of cord blood EV analysis [7,14]. To eliminate the possible effect of spontaneous delivery on the number of detected EVs [11,12], all patients included in the study were born by cesarean section. Due to ethical reasons, the sampling of cord blood was carried out after the manual removal of the placenta. It is not known if such approach affects the counts of detected EVs more than the inherently less safe sampling of blood before the umbilical cord clamping. Recently, we described that the delay in cord blood processing leads to a significant increase in the platelet EV count, while their count in platelet free plasma is reasonably stable [14]. Cord blood samples in our study were processed right away, and the platelet free plasma was flash frozen in liquid nitrogen. The freezing of samples for EV analysis is a controversial issue [26], but it is often the only option to overcome logistical hurdles of study set up in the clinical settings. Care was taken to process the samples in both experimental cohorts identically. To prevent potential operator bias, the samples were blinded and their nature revealed after the completion of the data analysis.

Flow cytometry of EVs is straightforward; however, it must be carefully controlled. We consistently utilized standard size calibration beads to set the limits of the EVs acquisition gate. The buffer used for the sample preparation was filtered to lower the background noise. The antibodies utilized for EV labeling were titrated to be used at saturating concentration [27]. To increase the specificity of EV detection, only the EVs simultaneously positive for two independent phenotype markers were analyzed. This approach limits the number of nonspecifically labeled particles detected, as demonstrated by very low numbers of double positive events in the IgG isotype controls. The markers utilized in the study for the detection of platelet, activated platelet, and endothelial EVs are all well characterized and were used in previous flow cytometry EV studies [28,29].

The presence of EVs in cord blood platelet free plasma was verified by immune electron microscopy. In accordance with previous reports, the EVs appeared as “cup-shaped” particles mostly with size around 200 nm [30]. Importantly, the labeling with the CD41 MAb followed by colloidal gold secondary antibody clearly marked some of the visualized EVs, indicating that the signal detected by flow cytometry was not artificial. The acquisition rate during the sample analysis was below 500 events per second (median 219), which in our preliminary experiments limited coincident “swarm” EV detection, an unavoidable complication of EV analysis by conventional flow cytometers [8]. As the refractive index of the beads utilized for definition of the acquisition gate is higher than that of EVs, we estimated the real size of EVs detected in the gate using FCMPASS software utilizing predefined refractive index for large EVs [24]. The calculated size of EVs detected in the gate was between 400 and 2000 nm. While the calculated upper limit of the gate seems that it may accommodate platelets, we previously showed that the signal of intact platelets is higher and outside the gate [14].

The size of EVs is typically described to be in the range 50–1000 nm. A recent study utilizing nanoparticle tracking analysis and electron microscopy estimated that only about 1% of EVs in blood plasma have a diameter above 300 nm [31]. This indicates that our study and all other studies of EVs utilizing conventional flow cytometry instruments are analyzing only a small subset of large EVs, while the rest of the present EVs are below the instrument detection limit [8]. The current set of recommendations for EV flow cytometry experiments [32] includes fluorescence calibration, which allows reproduction of the experiments with instruments differing in fluorescence sensitivity. Our study lacks fluorescence calibration, as it was accomplished before the recommendations were published. It should be noted that the fluorescence calibration does not have any effect on the study results.

### 4.3. Study Results

To control the reproducibility of our analytical procedure, all patient samples were analyzed in two separately labeled aliquots. The variation of the results between the aliquots was negligible in three quarters of the samples. The variability in the other quarter of the samples was acceptable, providing highly concordant data. The average count of the detected large EVs was below 1000 EVs/µL of plasma for all studied EVs phenotypes and study groups, suggesting their low incidence in comparison with normal counts of blood cells (e.g., 250,000 platelets/µL). Our initial hypothesis was that preterm newborns might differ in counts of platelet and endothelial EVs due to known gestational age dependent discrepancies in both cell lineages [15,25]. Despite this, the counts of the studied EVs were similar in both experimental groups, and there was no obvious correlation of the EV counts with gestational age. Similarly, we reasoned that underlying pathologies associated with preterm birth might be associated with notable changes in platelet and endothelial EV counts, but comparison with the healthy control group again did not provide support for our assumption. This result, however, must be interpreted with caution as the preterm group was heterogeneous and the numbers of distinct pathologies low. In addition, all mothers in the preterm group were given antenatal prophylactic corticosteroids with unknown effect on the presence of EVs in newborn blood.

During the visual inspection of frozen plasma samples, we noted that some of them displayed signs of hemolysis. Spectrophotometric measurements confirmed levels of free hemoglobin above 0.1 mg/mL in seven plasma samples. The counts of platelet and activated platelet EVs in hemolytic samples were notably higher. Hemolysis is known to promote platelet activation both in vivo and in vitro, which may explain increased counts of platelet EVs in the hemolytic samples [33]. Out of seven hemolytic samples, six were present in the preterm group and one in the control group, which is suggestive of the hemolysis association with preterm birth, reflecting the ongoing pathophysiologic events, but the difference was not statistically significant, perhaps due to small size of the experimental groups. An alternative explanation of the hemolysis presence is an unrecognized preanalytical error leading to in vitro hemolysis [34]. The count of endothelial EVs was also moderately increased. The potential improper sample handling should not have any effect on the count of endothelial EVs as vascular endothelium is not present in vitro.

In our previous study with fresh cord blood plasma samples, we noted a lower median fluorescence intensity of platelet large EVs after the labeling with CD36 or CD62 MAb in a small group of preterm newborns [14]. In our current study with frozen samples, there was no significant difference in the fluorescence intensity of the studied EVs except for a modestly decreased fluorescence of CD31-labeled EVs in the preterm group. Gestation age dependent expression of several endothelial receptors has been reported, but the level of CD31 on endothelial cells seemed to be gestation age insensitive [35]. There was no difference in the fluorescence intensity of large EVs in the hemolytic and nonhemolytic samples, suggesting that the identified differences in the EV counts were not caused by diverse expression of the phenotypic markers.

## 5. Conclusions

Our study confirmed the presence of large EVs of platelet and endothelial origin in cord blood plasma of preterm and control newborns using conventional flow cytometry. The size of the detected EVs was in the range 400–2000 nm and their average count was below 1000/µL of plasma regardless their phenotype. Differences in the counts of platelet, activated platelet, and endothelial large EVs between the preterm and control groups were not significant, and the counts of the EVs did not display any gestation age-dependent trend. Hemolytic samples had significantly elevated levels of large EVs, implying that the monitoring of free hemoglobin concentration should be integral part of similar studies. Future research utilizing improved flow cytometers allowing detection of more representative segment of EVs [36,37] is needed to clarify if cord blood plasma quantitative EVs immunophenotyping has possible diagnostic and prognostic potential in diverse neonatal complications.

## Figures and Tables

**Table 1:** Description of the experimental groups.

	Study Group (*n* = 20) PRTM	Control Group (*n* = 10) CTRL	*p* Value
Gestational age, wk, mean ± SD	31.5 ± 2.0	38.1 ± 1.8	0.0001
Birth weight, g, mean ± SD	1609 ± 437	3128 ± 349	0.0001
FIRS, *n* (%)	7 (35)	0 (0)	0.06
PPROM, *n* (%)	4 (20)	0 (0)	0.27
FGR, *n* (%)	8 (40)	0 (0)	0.03
M/F ratio (%)	60/40	20/80	0.06
Composite morbidity, *n* (%)	3 (15)	0 (0)	N/A

Abbreviations: FIRS, fetal inflammatory response syndrome; FGR, fetal growth restriction; M/F, male/female ratio; PPROM, preterm premature rupture of membrane.

**Table 2:** EV counts in PRTM and CTRL presented as a mean ± SD. Differences between PRTM and CTRL groups were not significant.

EVs	PRTM EVs/µL	CTRL EVs/µL	*p* Value
CD41+/CD36+	914 ± 654	756 ± 670	0.3789
CD41+/CD62+	435 ± 369	314 ± 349	0.3671
CD31+/CD105+	475 ± 341	564 ± 792	0.3442

**Table 3:** EV counts of hemolytic (H+) and non-hemolytic (H−) cord blood plasma samples presented as a mean ± SD.

EVs	H− EVs/µL	H+ EVs/µL	*p* Value
CD41+/CD36+	558 ± 294	1860 ± 607	<0.001
CD41+/CD62+	232 ± 168	928 ± 370	<0.001
CD31+/CD105+	383 ± 345	906 ± 872	<0.05

## Data Availability

The data used to support the findings of the presented study are available from the corresponding author upon request.
